# Supplementary material for: Detailed Studies on the Methoxylation and Subsequent Dealkylation of N,N-Diethylbenzenesulfonamide Using a Tailor-Made Electrosynthetic Reactor
Source: Molecules. 2024 Nov 21;29(23):5496. doi: 10.3390/molecules29235496 (PMC11643773; doi:10.3390/molecules29235496)
Supplement: Supplementary file 1 [file molecules-29-05496-s001.zip › molecules-3268942-supplementary.pdf]

Supplementary Materials

Detailed studies on the methoxylation and subsequent dealkylation of *N,N*-diethylbenzenesulfonamide using a tailor-made electrosynthetic reactor

Ernák F. Várda [1], Imre Gyűjtő [1,2], Ferenc Ender [3,4], Richárd Csekő [3,4], György T. Balogh [1,5,6,*], and Balázs Volk [1,2,*]

[1] Department of Chemical and Environmental Process Engineering, Budapest University of Technology and Economics, Műegyetem rakpart 3, H-1111 Budapest, Hungary; varda.ernakferenc@edu.bme.hu (E.F.V.); gyujtoimre@gmail.com (I.G.)

[2] Egis Pharmaceuticals Plc., Directorate of Drug Substance Development, P.O. Box 100, H-1475 Budapest, Hungary; volk.balazs@egis.hu (B.V.)

[3] Department of Electron Devices, Budapest University of Technology and Economics, Műegyetem rakpart 3, H-1111 Budapest, Hungary; ender.ferenc@vik.bme.hu (F.E.); csekorichard@edu.bme.hu (R.C.)

[4] Spinsplit Research and Development Ltd., Szőlőskert u. 0182/135, H-2220 Vecsés, Hungary

[5] Department of Pharmaceutical Chemistry, Semmelweis University, Hőgyes Endre út 9, H-1092 Budapest, Hungary; balogh.gyorgy.tibor@semmelweis.hu (G.T.B.)

[6] Center for Pharmacology and Drug Research & Development, Department of Pharmaceutical Chemistry, Semmelweis University, Budapest, Hungary

*Correspondence: balogh.gyorgy.tibor@semmelweis.hu (G.T.B.); volk.balazs@egis.hu (B.V.)

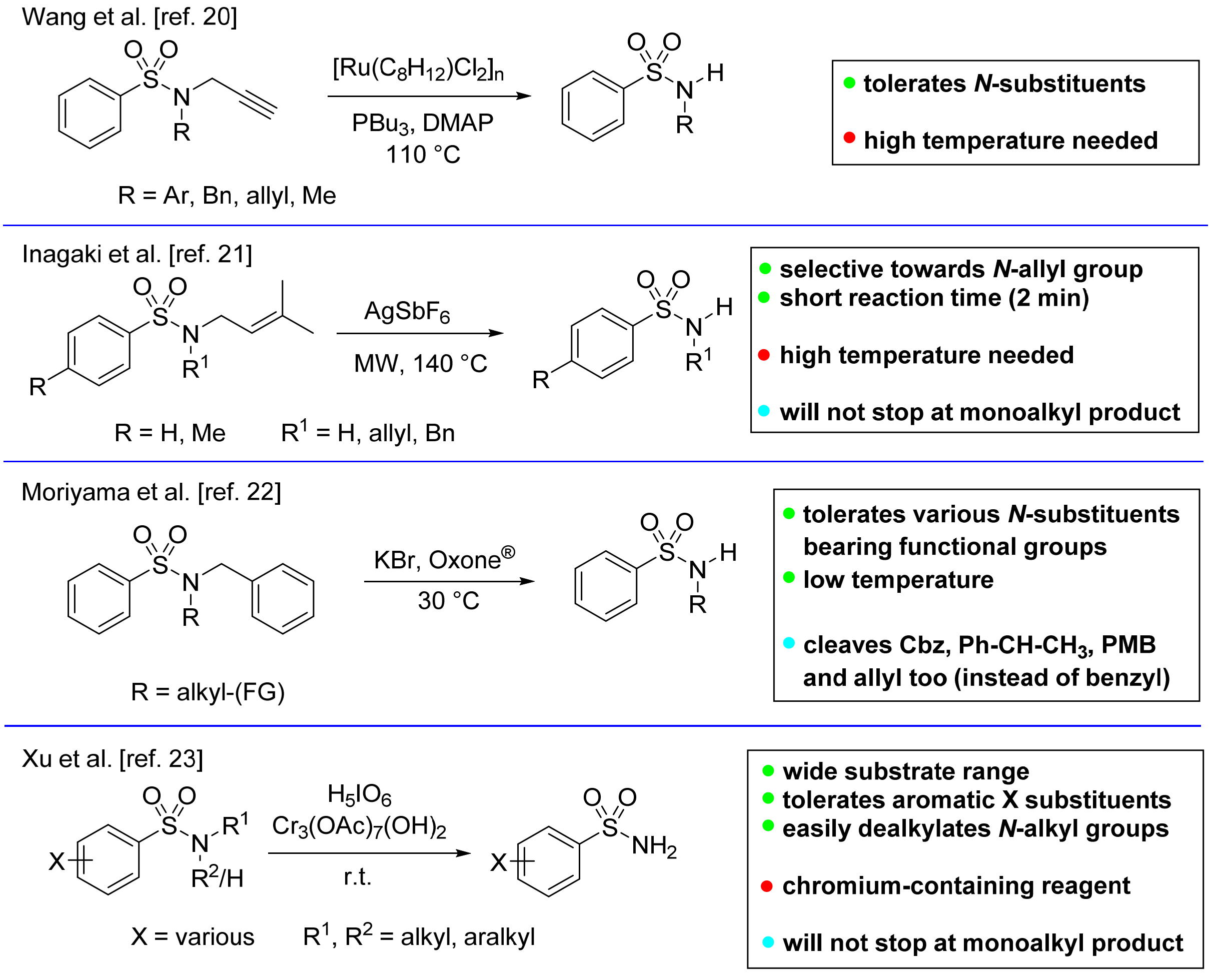

Scheme S1. Methods for the chemical dealkylation of *N*-alkyl- or *N,N*-dialkylbenzenesulfonamides with advantages (green), disadvantages (red) and further characteristics (blue)

Figure S1. Schematic view of the tailor-made electrochemistry potentiostat

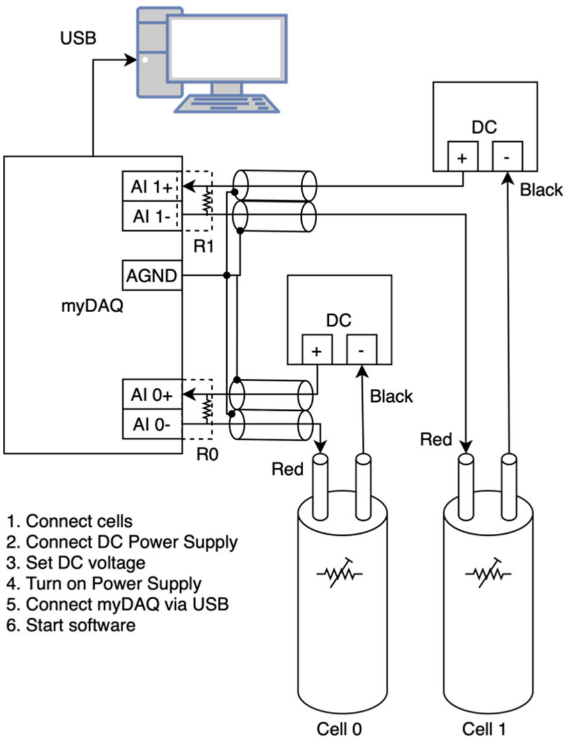

Figure S2. Representative photo of the experimental setup of the tailor-made electrochemical station

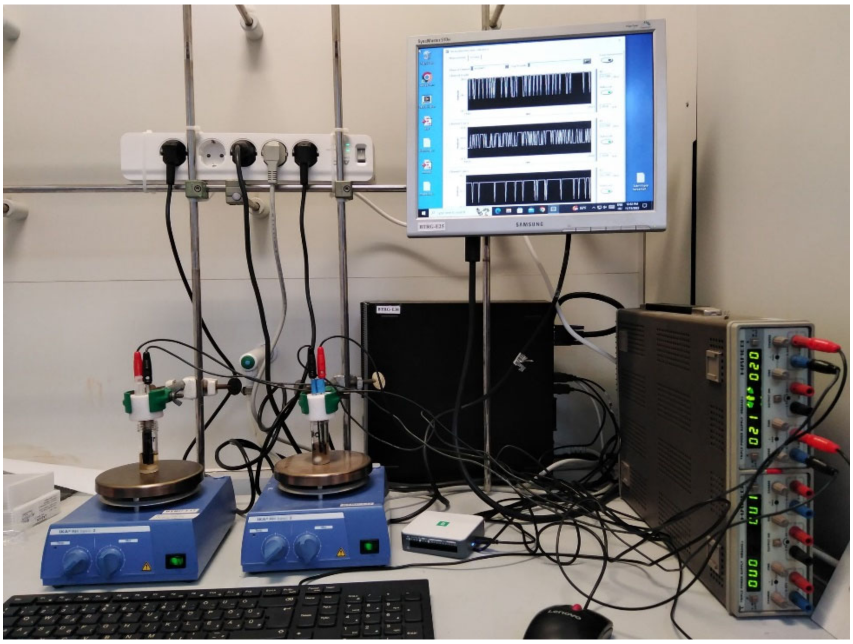

Figure S3. Circuit diagram of a single cell

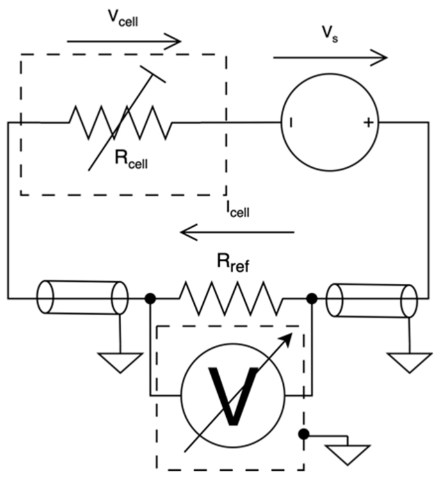

Electrochemistry potentiostat was assembled in-house as depicted in Fig. S1–S3. The current measurement setup consists of two electrochemical cells (Cell 0 and Cell 1), however the design is scalable to accommodate more cells. Each cell consists of two electrodes where each one electrode is connected to the negative pole of a current limited voltage source (Hameg Triple Power Supply) and the other one is connected to a nonreferenced differential voltage monitor (National Instruments myDAQ, NI, Austin, Texas). Shielded coax cables were used to reduce induced noise and the shields were connected to the common analog ground of the voltage monitor. Therefore, each cell forms an electrical circuit as depicted in Fig. S3.

The cell voltage and current are given as

$$V_{cell} = V_s - R_{ref}\, I_{cell} \quad \text{(S1)}$$

$$I_{cell} = v/R_{ref} \quad \text{(S2)}$$

respectively, where *V* is the differential voltage drop measured on the 10 Ω ± 1% R_{ref} resistor and V_s is the source voltage set on the voltage generator. For each consecutive measurement 1000 samples were taken with 1 ks/sec sample rate and averaged, then the cell current was expressed using Eq. S2.

Table S1. Screening of electrical input, starting material concentration, electrode type, charge, solvent and electrolyte concentration[a]

Time [min]	Electrical input [mA]	Starting material [mg]	Elect-rodes	Q [F/mol]	Solvent	Electrolyte	Relative quantity [%] by HPLC/UV (λ=220 nm)			
							1	2a	3	other[b]
12	100	35.2	RVC	4.5	CH_3CN/MeOH 9:1	TBAP 0.500 M	3	55	9	33
24	50	35.2	RVC	4.5	CH_3CN/MeOH 9:1	TBAP 0.500 M	8	68	10	14
40	30	35.2	RVC	4.5	CH_3CN/MeOH 9:1	TBAP 0.500 M	19	54	8	19
60	20	35.2	RVC	4.5	CH_3CN/MeOH 9:1	TBAP 0.500 M	28	43	13	16
120	10	35.2	RVC	4.5	CH_3CN/MeOH 9:1	TBAP 0.500 M	68	-	13	19
60	20	35.2	G[c]	4.5	MeOH	TBAP 0.500 M	-	77	6	17
40	20	35.2	G	3.0	MeOH	TBAP 0.500 M	12	67	7	14
40	20	35.2	G	3.0	MeOH	TBAP 0.500 M	9	77	6	8
31	20	35.2	G	2.3	MeOH	TBAP 0.500 M	20	73	-	7
49	20	35.2	G	3.7	MeOH	TBAP 0.500 M	1	49	2	48
27	30	35.2	G	3.0	MeOH	TBAP 0.500 M	18	53	15	14
60	20	35.2	G	4.5	CH_3CN/MeOH 9:1	TBAP 0.500 M	1	46	14	39
31	20	35.2	G	2.3	CH_3CN/MeOH 9:1	TBAP 0.500 M	12	66	11	11
31	20	35.2	G	2.3	CH_3CN/MeOH 9:1	TBAP 0.500 M	16	62	11	11
40	20	35.2	G	3.0	CH_3CN/MeOH 9:1	TBAP 0.500 M	2	74	11	13
36	20	35.2	G	2.7	CH_3CN/MeOH 9:1	TBAP 0.500 M	22	41	22	15
60	20	35.2	G	4.5	MeOH	Bu_4NBF_4 0.500 M	6	78	2	14
60	20	35.2	RVC	4.5	CH_3CN/MeOH 9:1	Bu_4NBF_4 0.500 M	77	16	1	6
31	20	35.2	G	2.3	MeOH	Bu_4NBF_4 0.500 M	47	12	-	41
40	30	35.2	G	4.5	MeOH	Bu_4NBF_4 0.500 M	44	50	2	4
60	20	35.2	G	4.5	CH_3CN/MeOH 9:1	Bu_4NBF_4 0.500 M	1	64	5	30
49	20	35.2	G	3.7	CH_3CN/MeOH 9:1	Bu_4NBF_4 0.500 M	3	59	5	33

150	20	35.2	G	11.25	MeOH	Bu_4NBF_4 0.500 M	-	76	7	17
34	20	20	G	4.5	MeOH	TBAP 0.500 M	11	59	21	9
34	20	20	G	4.5	CH_3CN/MeOH 9:1	TBAP 0.500 M	3	35	22	40
85	20	50	G	4.5	CH_3CN/MeOH 9:1	TBAP 0.500 M	2	44	39	15
85	20	50	G	4.5	MeOH	TBAP 0.500 M	13	48	39	-

[a] Reactor volume: 10 mL, solvent volume: 6 mL, ambient temperature. [b] Total amount of unidentified compounds. Compounds **4** and **5** were not detected. [c] Graphite.

Table S2. Screening of electrical input and reaction time, and comparison of the commercially available IKA reactor with our tailor-made model (I=20 mA)[a]

Q [F/mol]	Time [min]	Compound	Relative quantity [%] by HPLC/UV (λ=220 nm)		
			tailor-made	tailor-made	IKA
0.8	10	**1**	74	74	69
		**2a**	24	25	29
		**3**	2	1	2
1.5	20	**1**	50	49	44
		**2a**	47	47	52
		**3**	3	4	4
2.3	30	**1**	30	31	26
		**2a**	65	65	69
		**3**	5	4	5
3.0	40	**1**	20	18	18
		**2a**	76	77	77
		**3**	4	5	5
3.8	50	**1**	15	12	15
		**2a**	80	82	79
		**3**	5	6	6
4.5	60	**1**	16	14	15
		**2a**	78	81	80
		**3**	6	5	5

[a] Reactor volume: 10 mL, solvent (MeOH) volume: 6 mL, ambient temperature, electrolyte: 0.167 M TBAP, electrodes: graphite, starting material: 35.2 mg (27.5 mM), solvent: MeOH, electrical input: 20 mA.

Table S3. Screening of electrical input and reaction time, and comparison of the commercially available IKA reactor with our tailor-made model (I=50 mA)[a]

Q [F/mol]	Time [min]	Compound	Relative quantity [%] by HPLC/UV (λ=220 nm)		
			tailor-made	tailor-made	IKA
1.5	8	**1**	58	51	63
		**2a**	40	46	35
		**3**	2	3	2
3.0	16	**1**	32	23	36
		**2a**	63	72	60
		**3**	5	5	4
4.5	24	**1**	22	19	20
		**2a**	73	76	75
		**3**	5	5	5
6.0	32	**1**	27	25	22
		**2a**	68	70	73
		**3**	5	5	5

[a] Reactor volume: 10 mL, solvent (MeOH) volume: 6 mL, ambient temperature, electrolyte: 0.167 M TBAP, electrodes: graphite, starting material: 35.2 mg (27.5 mM), solvent: MeOH, electrical input: 50 mA.

Table S4. Screening of electrical input and reaction time, and comparison of the commercially available IKA reactor with our tailor-made model (I=100 mA)[a]

Q [F/mol]	Time [min]	Compound	Relative quantity [%] by HPLC/UV (λ=220 nm)		
			tailor-made	tailor-made	IKA
1.5	4	**1**	63	80	70
		**2a**	34	19	28
		**3**	3	1	2
3.0	8	**1**	47	65	50
		**2a**	51	32	46
		**3**	2	3	4
4.5	12	**1**	40	57	39
		**2a**	56	40	56
		**3**	4	3	5
6.0	16	**1**	39	53	39
		**2a**	57	43	57
		**3**	4	4	4

[a] Reactor volume: 10 mL, solvent (MeOH) volume: 6 mL, ambient temperature, electrolyte: 0.167 M TBAP, electrodes: graphite, starting material: 35.2 mg (27.5 mM), solvent: MeOH, electrical input: 100 mA.

Table S5. Effect of 1% H_2O on the outcome of the reaction[a]

Electrical input [mA]	Q [F/mol]	Solvent	Time [min]	Relative quantity [%] by HPLC/UV (λ=220 nm)			
				**1**	**2a**	**3**	other[b]
20	0.8	MeOH	10	74	24	2	-
20	1.5	MeOH	20	50	46	4	-
20	2.3	MeOH	30	30	65	5	-
20	3.0	MeOH	40	20	76	4	-
20	3.8	MeOH	50	15	80	5	-
20	4.5	MeOH	60	16	78	5	1
20	5.3	MeOH	70	18	76	6	-
50	1.5	MeOH	8	58	40	2	-
50	3.0	MeOH	16	33	63	4	-
50	4.5	MeOH	24	22	73	5	-
100	1.5	MeOH	4	63	34	3	-
100	3.0	MeOH	8	46	50	4	-
100	4.5	MeOH	12	40	56	4	-
100	6.0	MeOH	16	39	57	4	-
20	1.1	MeOH/H_2O 99:1	15	76	19	5	-
20	2.3	MeOH/H_2O 99:1	30	52	32	16	-
20	3.4	MeOH/H_2O 99:1	45	35	53	12	-
20	4.5	MeOH/H_2O 99:1	60	48	35	17	-

[a] The experiments were carried out in our tailor-made reactor, the amount of **1** was 35.2 mg, graphite electrodes were applied, and the electrolyte was TBAP (0.167 M). [b] Total amount of unidentified compounds. Compounds **4** and **5** were not detected.

Table S6. Comprehensive solvent screening[a]

Q [F/mol]	Solvent	Time [min]	Relative quantity [%] by HPLC/UV (λ=220 nm)				
			**1**	**2a/2b/2c**	**3**	**5**	**other**[b]
2.3	MeOH/H_2O 95:5	30	43	53 (**2a**)	-	-	4
4.5	MeOH/H_2O 95:5	60	17	71 (**2a**)	-	-	12
9.0	MeOH/H_2O 95:5	120	16	52 (**2a**)	12	-	20
2.3	CH_3CN/MeOH 9:1	30	19	45 (**2a**)	33	-	3
4.5	CH_3CN/MeOH 9:1	60	-	33 (**2a**)	53	-	14
9.0	CH_3CN/MeOH 9:1	120	-	- (**2a**)	22	52	26
2.3	CH_3CN/IPA 9:1	30	13	36 (**2b**)	32	-	19
4.5	CH_3CN/IPA 9:1	60	-	12 (**2b**)	30	41	17
9.0	CH_3CN/IPA 9:1	120	-	- (**2b**)	27	53	20
2.3	CH_3CN/IPA/H_2O 89:10:1	30	15	16 (**2b**)	43	11	15
4.5	CH_3CN/IPA/H_2O 89:10:1	60	-	3 (**2b**)	50	31	16
9.0	CH_3CN/IPA/H_2O 89:10:1	120	-	- (**2b**)	27	72	1
2.3	CH_3CN/IPA/H_2O 85:10:5	30	-	- (**2b**)	82	-	18
4.5	CH_3CN/IPA/H_2O 85:10:5	60	-	- (**2b**)	89	11	0
9.0	CH_3CN/IPA/H_2O 85:10:5	120	-	- (**2b**)	69	31	0
2.3	CH_3CN/*t*-BuOH 9:1	30	54	- (**2c**)	46	-	0
4.5	CH_3CN/*t*-BuOH 9:1	60	64	- (**2c**)	36	-	0
9.0	CH_3CN/*t*-BuOH 9:1	120	-	- (**2c**)	-	-	100[c]
2.3	CH_3CN/MeOH/H_2O 89:10:1	30	46	47 (**2a**)	7	-	0
4.5	CH_3CN/MeOH/H_2O 89:10:1	60	13	78 (**2a**)	8	-	0
9.0	CH_3CN/MeOH/H_2O 89:10:1	120	-	- (**2a**)	-	-	100[c]
2.3	CH_3CN/MeOH/H_2O 85:10:5	30	29	63 (**2a**)	8	-	0
4.5	CH_3CN/MeOH/H_2O 85:10:5	60	10	81 (**2a**)	9	-	0
9.0	CH_3CN/MeOH/H_2O 85:10:5	120	20	58 (**2a**)	9	-	13
2.3	CH_3CN/*t*-BuOH/H_2O 89:10:1	30	44	- (**2c**)	56	-	0
4.5	CH_3CN/*t*-BuOH/H_2O 89:10:1	60	8	- (**2c**)	82	10	0
9.0	CH_3CN/*t*-BuOH/H_2O 89:10:1	120	-	- (**2c**)	60	40	0
2.3	CH_3CN/*t*-BuOH/H_2O 85:10:5	30	55	- (**2c**)	45	-	0
4.5	CH_3CN/*t*-BuOH/H_2O 85:10:5	60	13	7 (**2c**)	72	8	0
9.0	CH_3CN/*t*-BuOH/H_2O 85:10:5	120	-	- (**2c**)	80	20	0

[a] Tailor-made reactor, solvent volume: 6 mL, amount of **1**: 35.2 mg (27.5 mM), electrodes: graphite, electrolyte: TBAP (0.167 M), electrical input: 20 mA. [b] Total amount of unidentified compounds. Compound **4** was not detected. [c] Dark color and several unidentified impurities.
